# INTERGENERATIONAL QUALITY OF LIFE: SIMILARITIES AND DIFFERENCES AMONG MUSICIANS

**DOI:** 10.1093/geroni/igae098.3502

**Published:** 2024-12-31

**Authors:** Whitney Nesser, Scott Snyder

**Affiliations:** Indiana State University, Terre Haute, Indiana, United States; University of Alabama at Birmingham, Birmingham, Alabama, United States

## Abstract

Music has been shown to improve Quality of Life (QoL) for various populations. However, few studies have studied older musicians’ QoL or QoL in an intergenerational sample of musicians. To address this gap this study assessed four QoL domains (physical, psychological, social, and environmental) in a nationally representative intergenerational sample of musicians using the WHOQOL-BREF scale. Data were obtained via a survey of professional and amateur musicians (N=407) across the United States (US). Results demonstrated that while the oldest subgroup of musicians had the highest mean scores for environmental, psychological and social QoL, there were no significant differences between age groups for any of the QoL domains. Rating scale responses for all QoL items were independent of age group except for “How often do you have negative feelings” (p=.01). Examination of standardized residual showed that significant effect was due to the older musicians responding “never” more frequently than expected. The Spearman correlations of environmental QoL with psychological, and social QoL were significantly lower (p <.05) in the older musicians (r=.442, r=.494) than in the aggregate of other age groups (r=.715, r=.733). Furthermore, there was a significantly lower Spearman correlation between “enjoyment of being a musician” and “impact of being a musician on QoL” for older respondents (r=.384) compared to younger respondents (r=.678). Future research is needed to establish normative QoL data for the US on all age groups as measured by the WHOQOL-BREF and to examine the role of music as a mediator of QoL across the lifespan.

